# Supplementary material for: Dynamic noise estimation: A generalized method for modeling noise fluctuations in decision-making
Source: bioRxiv. 2024 Jan 26:2023.06.19.545524. Preprint. [Version 2] doi: 10.1101/2023.06.19.545524 (PMC10849494; doi:10.1101/2023.06.19.545524)
Supplement: Supplement 1 [file NIHPP2023.06.19.545524v2-supplement-1.pdf]

bioRxiv preprint doi: https://doi.org/10.1101/2023.06.19.545524; this version posted January 26, 2024. The copyright holder for this preprint (which was not certified by peer review) is the author/funder, who has granted bioRxiv a license to display the preprint in perpetuity. It is made available under aCC-BY 4.0 International license.

Appendix A. Supplementary figures

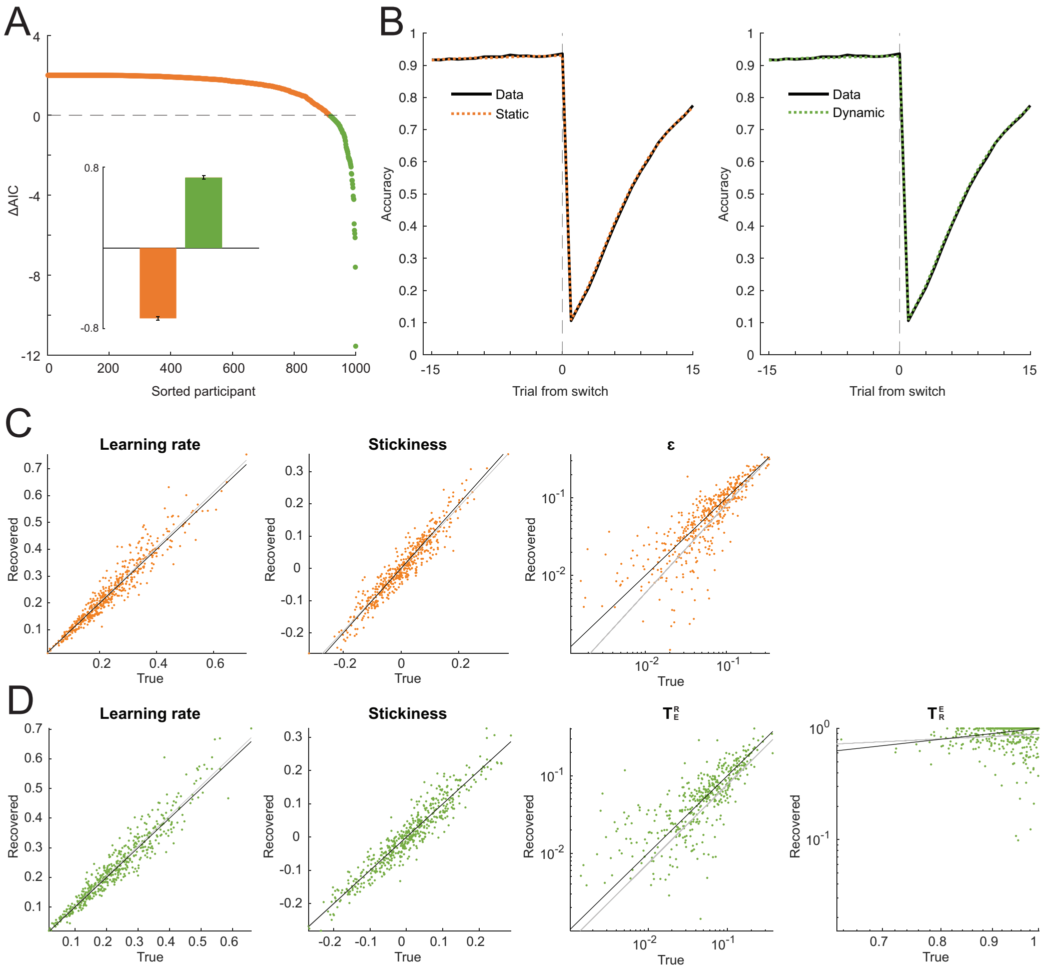

Figure A.7: **Both models with static and dynamic noise estimation can fully capture behavior and recover generative parameter values when the true model has static noise.** A: Evaluation of model fit with AIC on the data of 1,000 participants simulated using the static noise model. Each dot shows the difference in AIC for an individual between the static and dynamic models. A positive value (orange) indicates that the static model is favored and a negative value (green) means that the dynamic model is preferred by the criterion. The inset shows the mean difference in AIC between the models at the group level. B: Learning curves of both models and data. C: Parameter recovery using the static model. D: Parameter recovery using the dynamic model. For the dynamic equivalent of the static model, $T_E^R = \epsilon$ and $T_R^E = 1 - \epsilon$.

bioRxiv preprint doi: https://doi.org/10.1101/2023.06.19.545524; this version posted January 26, 2024. The copyright holder for this preprint (which was not certified by peer review) is the author/funder, who has granted bioRxiv a license to display the preprint in perpetuity. It is made available under aCC-BY 4.0 International license.

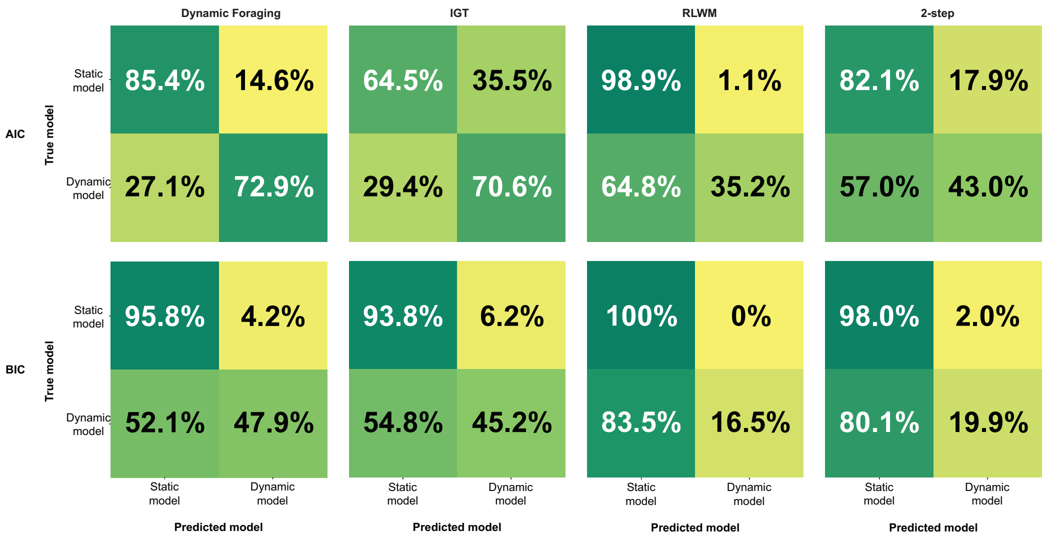

Figure A.8: **Model identification using AIC and BIC.** We performed model identification validation with confusion matrices [2]. To do so, we simulated data with parameters fitted to subjects' data. The AIC metric yielded better model identification than BIC. We note that simulations of the dynamic noise model were often mis-classified as being generated by the static noise model in RLWM and 2-step datasets. This is because most subjects in these datasets did not benefit substantially from dynamic noise estimation, and the parameters inferred made the dynamic noise model very similar to the static noise model. Thus, simulated behavior was in a range where both models were indistinguishable (since the static noise model is nested in the dynamic one). In these cases, the trivial improvements on likelihoods would be insufficient to offset the penalty incurred by the extra parameter in the dynamic model.

bioRxiv preprint doi: https://doi.org/10.1101/2023.06.19.545524; this version posted January 26, 2024. The copyright holder for this preprint (which was not certified by peer review) is the author/funder, who has granted bioRxiv a license to display the preprint in perpetuity. It is made available under aCC-BY 4.0 International license.

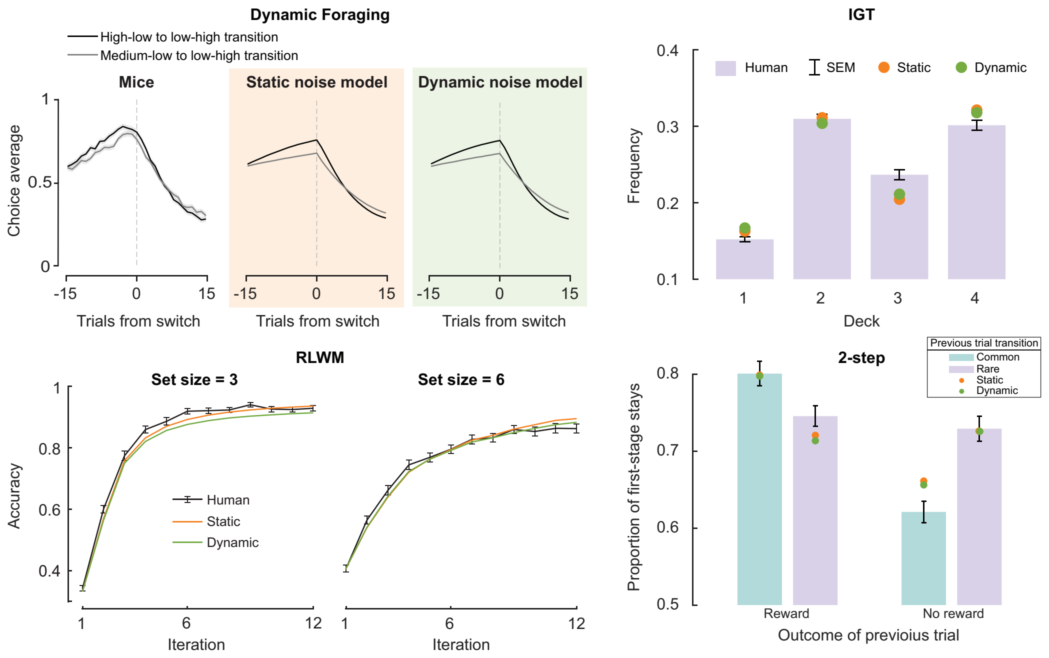

Figure A.9: **Model validation results on the empirical datasets.** Dynamic noise estimation did not alter the qualitative behavioral predictions made by the models.

bioRxiv preprint doi: https://doi.org/10.1101/2023.06.19.545524; this version posted January 26, 2024. The copyright holder for this preprint (which was not certified by peer review) is the author/funder, who has granted bioRxiv a license to display the preprint in perpetuity. It is made available under aCC-BY 4.0 International license.

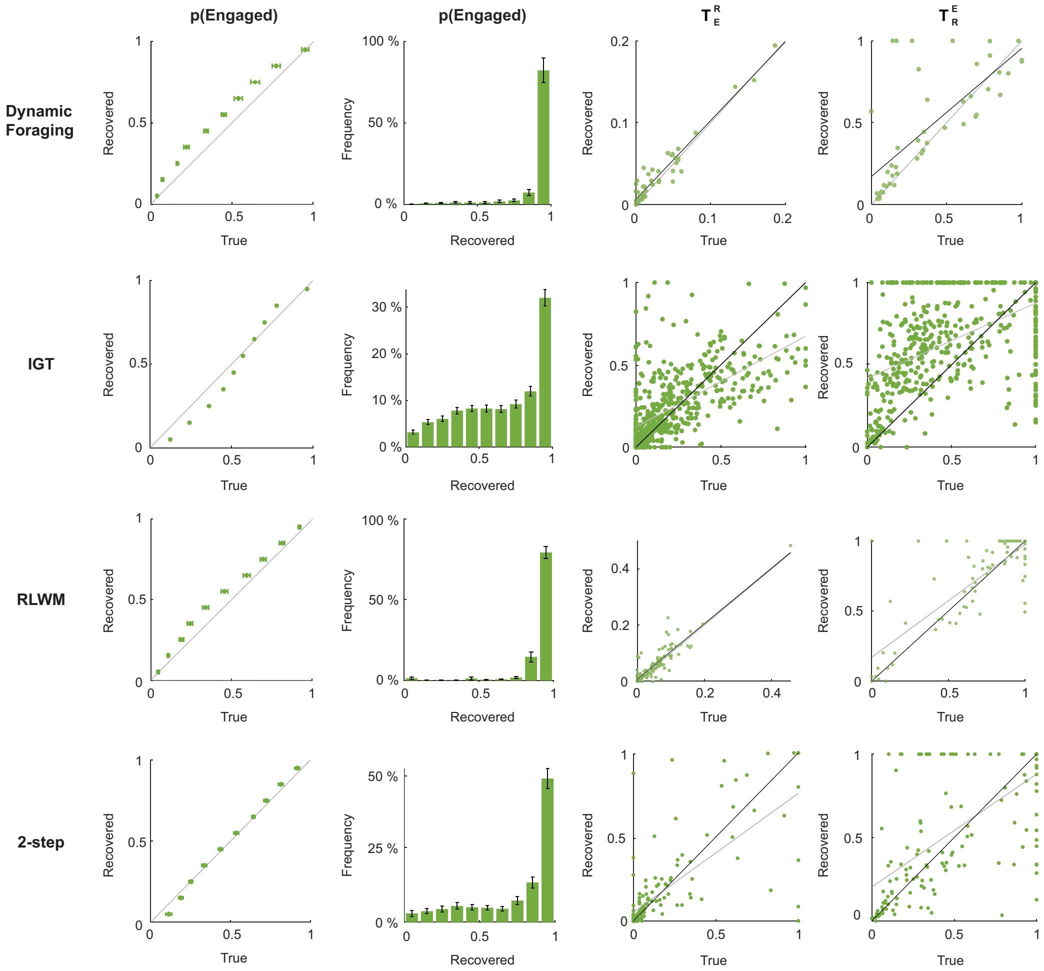

Figure A.10: **Recovery of latent state occupancy probability and noise parameters.** $p(Engaged)$ recovered well across datasets, with most recovered values between 0.9 and 1. T_E^R recovery was robust overall, while T_R^E recovered inadequately. This is because the lack of data in the random state led to insufficient potential transitions from the random to engaged state, which under-powered T_R^E recovery.

bioRxiv preprint doi: https://doi.org/10.1101/2023.06.19.545524; this version posted January 26, 2024. The copyright holder for this preprint (which was not certified by peer review) is the author/funder, who has granted bioRxiv a license to display the preprint in perpetuity. It is made available under aCC-BY 4.0 International license.

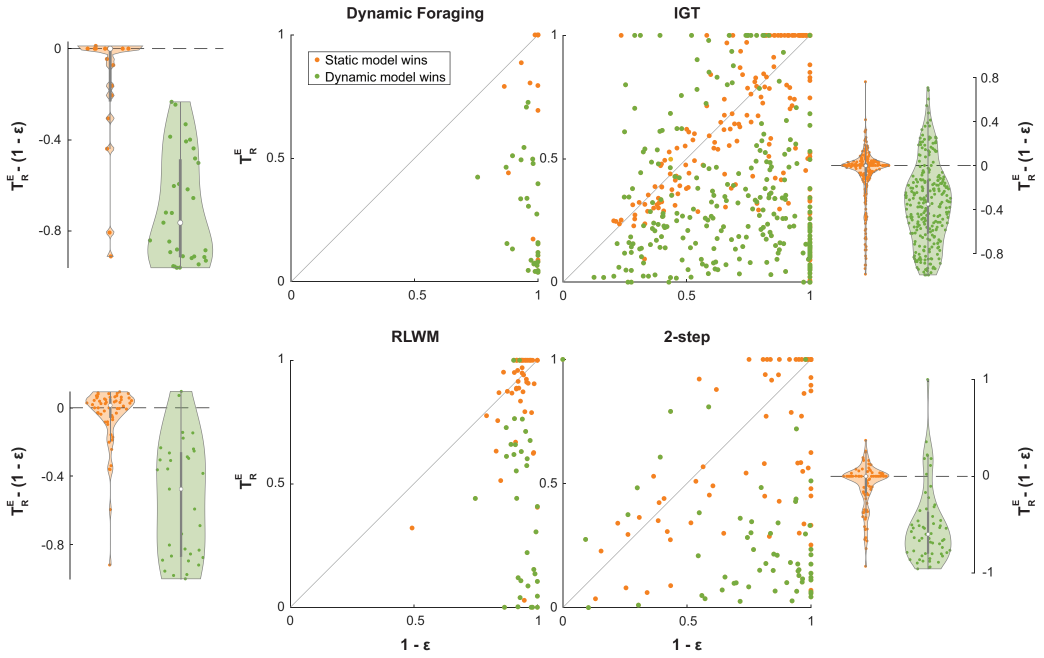

Figure A.11: **Improved fit by dynamic noise estimation is correlated to decreased estimation of the transition probability from the the random to engaged state.**

bioRxiv preprint doi: https://doi.org/10.1101/2023.06.19.545524; this version posted January 26, 2024. The copyright holder for this preprint (which was not certified by peer review) is the author/funder, who has granted bioRxiv a license to display the preprint in perpetuity. It is made available under aCC-BY 4.0 International license.

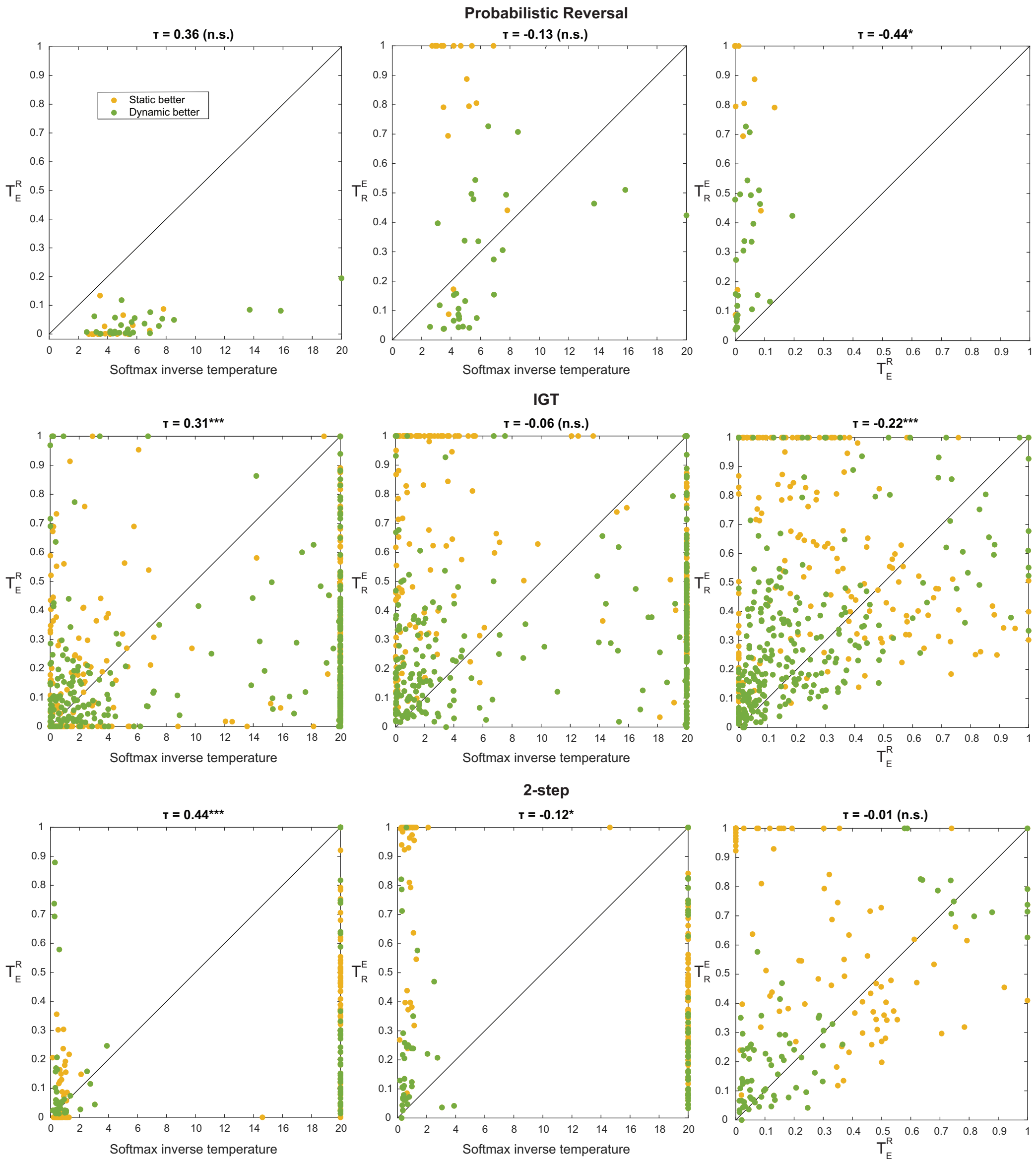

Figure A.12: **Relationships between noise parameters on the Dynamic Foraging [29], IGT [30], and 2-step [36] datasets.** No consistent correlations were found between the noise parameters including the softmax inverse temperature, T_E^R, and T_R^E.

bioRxiv preprint doi: https://doi.org/10.1101/2023.06.19.545524; this version posted January 26, 2024. The copyright holder for this preprint (which was not certified by peer review) is the author/funder, who has granted bioRxiv a license to display the preprint in perpetuity. It is made available under aCC-BY 4.0 International license.

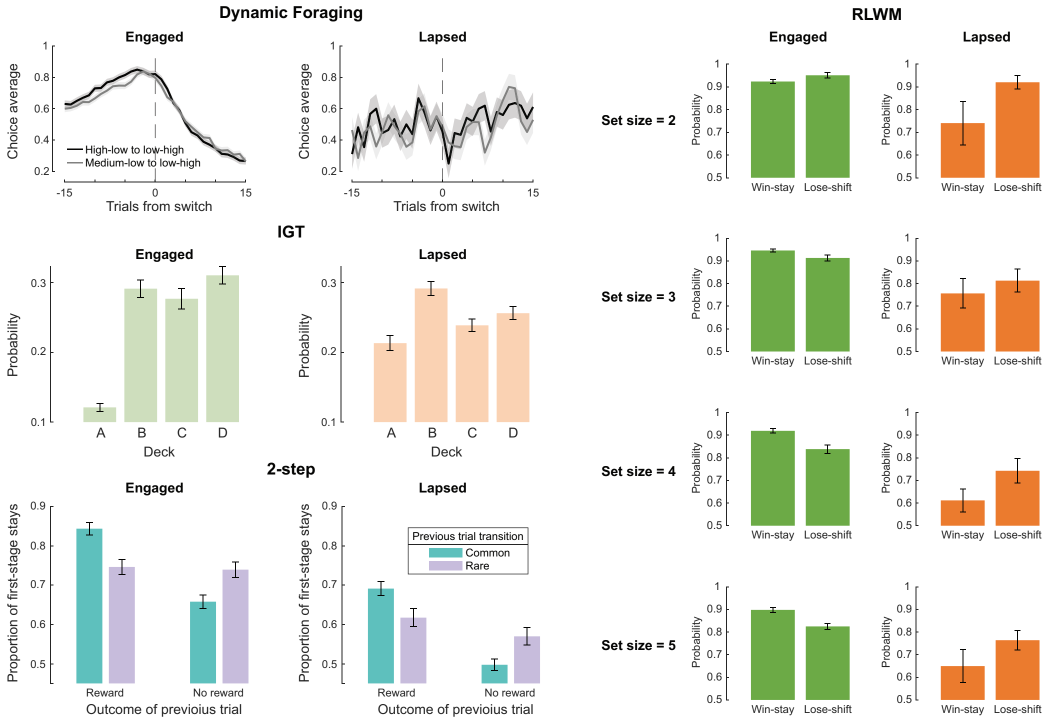

Figure A.13: **Behavior on putative engaged and lapsed trials predicted by dynamic noise estimation on the Dynamic Foraging [29], IGT [30], 2-step [33], and RLWM [4, 5] datasets.** On Dynamic Foraging, the learning curves around switches appear random-like during putative lapses. On the IGT dataset, choice frequencies of decks A and D regressed to the random level (one-tailed Wilcoxon signed-rank test $p = 9.35 \times 10^{-20}$ for A, $p = 0.48$ for B, $p = 0.11$ for C, and $p = 2.83 \times 10^{-5}$ for D). For 2-step, the accuracy decreased for all trial types (one-tailed Wilcoxon signed-rank test $p = 1.73 \times 10^{-5}$ for common and rewarded previous trials, $p = 0.019$ for rare and rewarded previous trials, $p = 5.33 \times 10^{-4}$ for common and unrewarded previous trials, and $p = 0.002$ for rare and unrewarded previous trials). On the RLWM dataset, the win-stay probability decreased more than the lose-shift probability overall (set size of 2: $p = 0.056$ for win-stay and $p = 0.38$ for lose-shift; set size of 3: $p = 0.07$ for win-stay and $p = 0.092$ for lose-shift; set size of 4: $p = 2.9 \times 10^{-4}$ for win-stay and $p = 0.34$ for lose-shift; set size of 5: $p = 0.006$ for win-stay and $p = 0.28$ for lose-shift).

bioRxiv preprint doi: https://doi.org/10.1101/2023.06.19.545524; this version posted January 26, 2024. The copyright holder for this preprint (which was not certified by peer review) is the author/funder, who has granted bioRxiv a license to display the preprint in perpetuity. It is made available under aCC-BY 4.0 International license.

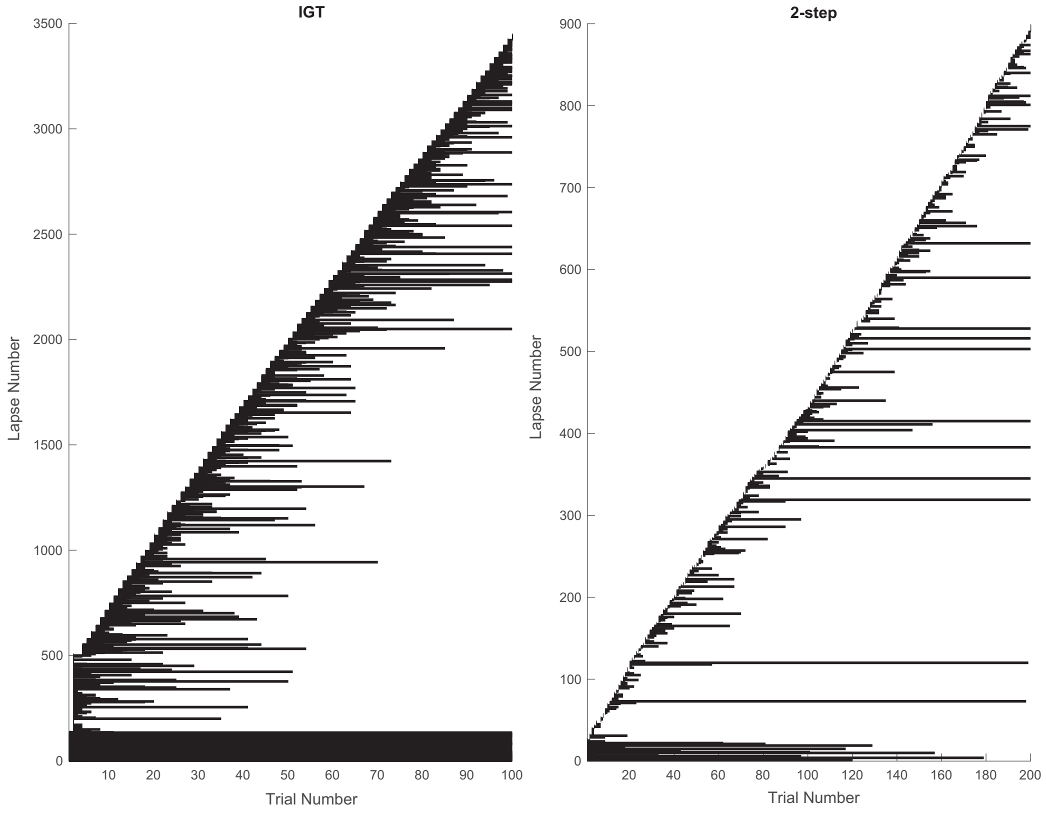

Figure A.14: **Putative lapses identified by dynamic noise estimation on the IGT [30] and 2-step [33] datasets, both with fixed numbers of trials across participants.** The lapses were identified as trials with $p(Engaged) < 0.5$, sorted by the start trial, and shown across participants.

bioRxiv preprint doi: https://doi.org/10.1101/2023.06.19.545524; this version posted January 26, 2024. The copyright holder for this preprint (which was not certified by peer review) is the author/funder, who has granted bioRxiv a license to display the preprint in perpetuity. It is made available under aCC-BY 4.0 International license.

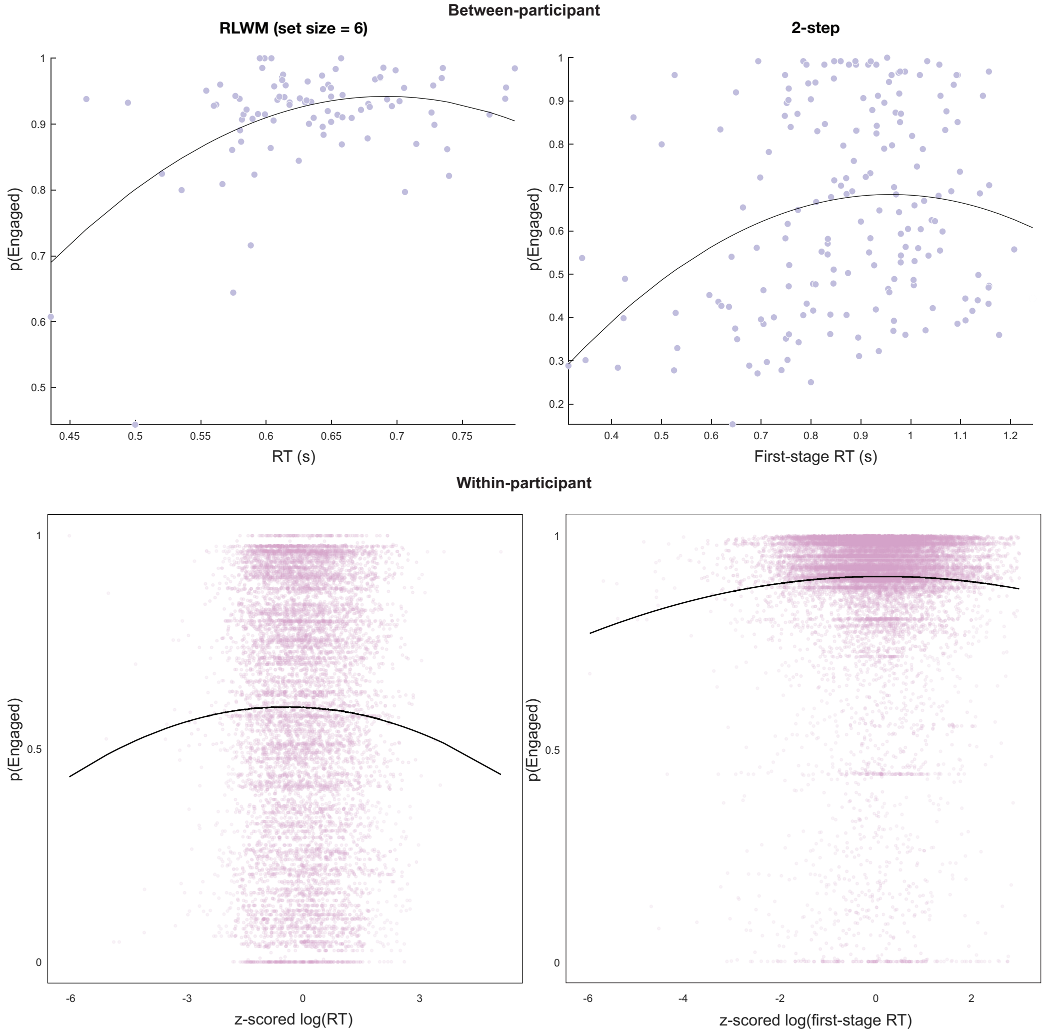

Figure A.15: **The inverted-U relationship between $p(Engaged)$ and reaction time between- and within-participants on the RLWM [32] and 2-step [36] datasets.** All p-values are less than 0.01 for the regression coefficients of the quadratic terms. The specific statistics are reported in Results.

bioRxiv preprint doi: https://doi.org/10.1101/2023.06.19.545524; this version posted January 26, 2024. The copyright holder for this preprint (which was not certified by peer review) is the author/funder, who has granted bioRxiv a license to display the preprint in perpetuity. It is made available under aCC-BY 4.0 International license.

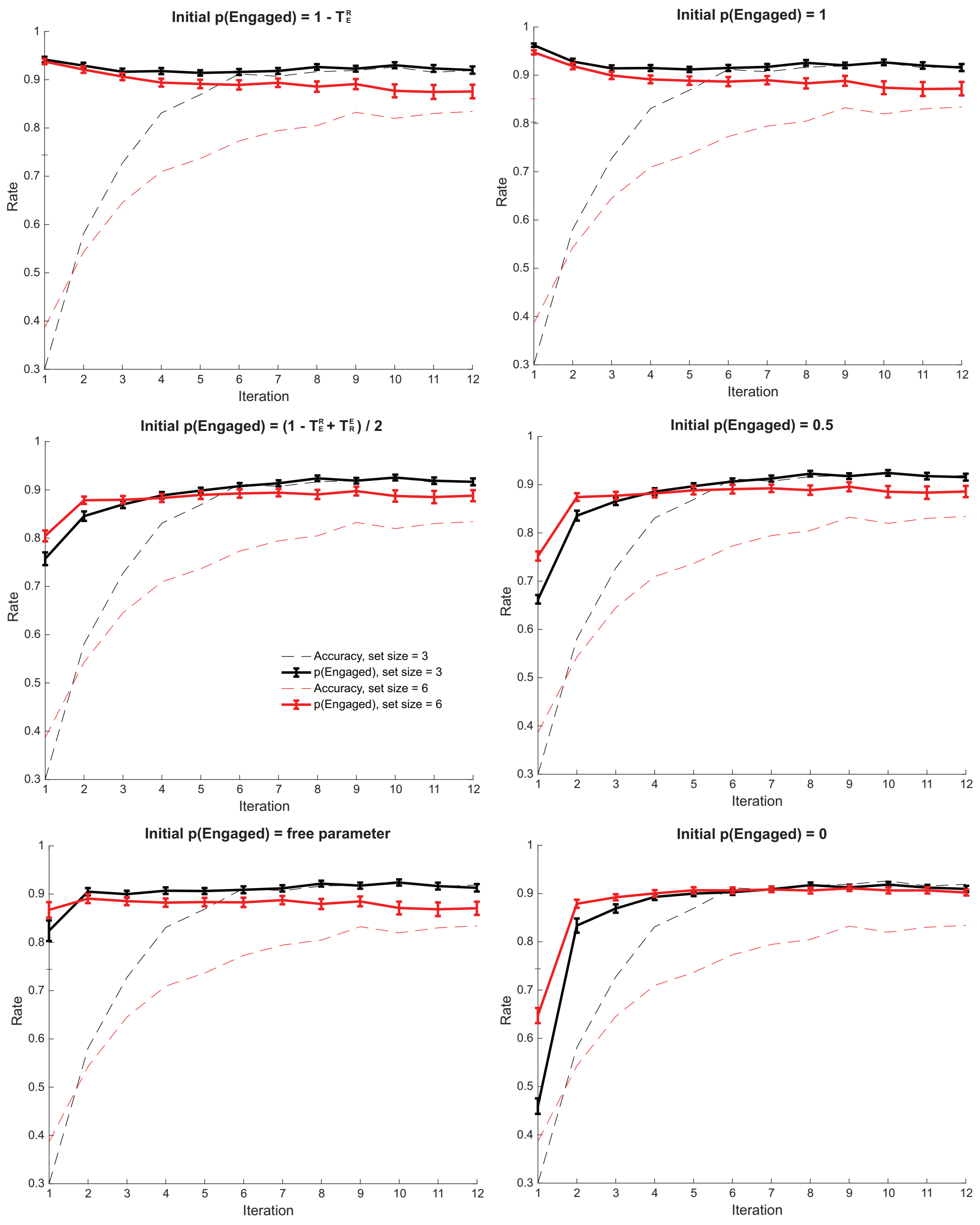

Figure A.16: **Different ways to initialize $p(Engaged)$ lead to different latent state occupancy estimations in the first few trials, but similar trajectories afterwards.** Note that the estimated engaged probability does not always follow the same trend as accuracy: towards the end of the block, while the difference in accuracy between set sizes of 3 and 6 shrinks, the difference in $p(Engaged)$ does not.

bioRxiv preprint doi: https://doi.org/10.1101/2023.06.19.545524; this version posted January 26, 2024. The copyright holder for this preprint (which was not certified by peer review) is the author/funder, who has granted bioRxiv a license to display the preprint in perpetuity. It is made available under aCC-BY 4.0 International license.

Appendix B. Model equations

Appendix B.1. Probabilistic Reversal

The model for the Probabilistic Reversal environment consists of 2 free parameters: α (learning rate) and ϕ (choice stickiness). The softmax inverse temperature is fixed at $\beta = 8$.

On trial t, the choice is made according to action probabilities computed through the softmax function. For example, the probability of choosing the left action is:

$$P_t(l) = \frac{1}{1 + \exp\Big(\beta \cdot \big(Q_t(r) - Q_t(l) - \phi \cdot \mathbb{1}_{a_{t-1}}[l]\big)\Big)},$$

where $\mathbb{1}_{a_{t-1}}[l]$ takes on the value of 1 if $a_{t-1} = l$ and -1 otherwise.

Once the reward r_t has been observed, the action values are updated:

$$Q_{t+1}(a_t) = Q_t(a_t) + \alpha \cdot (r_t - Q_t(a_t)).$$

Appendix B.2. Dynamic Foraging

The meta-learning model in the original paper was implemented [29]. The model has 7 parameters: β (softmax inverse temperature), $bias$ (for the right action), $\alpha_{(+)}$ (positive learning rate), $\alpha_{(-)_0}$ (baseline negative learning rate), α_v (rate of RPE magnitude integration), ψ (meta-learning rate for unexpected uncertainty), and ξ (forgetting rate).

On trial t, a decision is sampled from choice probabilities obtained through a softmax decision function applied to the action values of the left and right actions:

$$P_t(l) = \frac{1}{1 + \exp\Big(\beta \cdot \big(Q_t(r) - Q_t(l) + bias\big)\Big)}$$

and

$$P_t(r) = 1 - P_t(l).$$

Once the reward is observed, assuming the left action is chosen, its value is updated as follows:

$$Q_{t+1}(l) = Q_t(l) + \alpha_t \cdot \delta_t \cdot (1 - E_t),$$

bioRxiv preprint doi: https://doi.org/10.1101/2023.06.19.545524; this version posted January 26, 2024. The copyright holder for this preprint (which was not certified by peer review) is the author/funder, who has granted bioRxiv a license to display the preprint in perpetuity. It is made available under aCC-BY 4.0 International license.

where α_t is $\alpha_{(+)}$ if the reward-prediction error (RPE), $\delta_t = R_t - Q_t(l)$, is positive, and $\alpha_{(-)_t}$ otherwise. E_t is an evolving estimate of expected uncertainty calculated from the history of absolute RPEs:

$$E_{t+1} = E_t + \alpha_v \cdot v_t,$$

where

$$v_t = |\delta_t| - E_t.$$

When the RPE is negative, the negative learning rate is dynamically adjusted and lower-bounded by 0:

$$\alpha_{(-)_t} = \max\Big(0, \psi \cdot (v_t + \alpha_{(-)_0}) + (1 - \psi) \cdot \alpha_{(-)_{t-1}}\Big)$$

Finally, the unchosen action (e.g., right) is forgotten:

$$Q_{t+1}(r) = \xi \cdot Q_t(r).$$

Appendix B.3. IGT

The Value plus Sequential Exploration model [31] was implemented for the IGT dataset. The model is defined by 5 parameters: α (learning rate), β (softmax inverse temperature), θ (value sensitivity), Δ (decay), and ϕ (exploration bonus).

On trial t, the decision is sampled based on the probability of choosing deck d:

$$P_t(d) = \frac{\exp\Big(\beta \cdot \big(Explore_t(d) + Exploit_t(d)\big)\Big)}{\sum_{i=1}^{4} \exp\Big(\beta \cdot \big(Explore_t(i) + Exploit_t(i)\big)\Big)},$$

where $Explore_t(d)$ and $Exploit_t(d)$ are the action values of deck d using the exploration and exploitation weights. For the selected deck, their values are updated according to the following equations:

$$Explore_{t+1}(d) = 0$$

bioRxiv preprint doi: https://doi.org/10.1101/2023.06.19.545524; this version posted January 26, 2024. The copyright holder for this preprint (which was not certified by peer review) is the author/funder, who has granted bioRxiv a license to display the preprint in perpetuity. It is made available under aCC-BY 4.0 International license.

and

$$Exploit_{t+1}(d) = \Delta \cdot Exploit_t(d) + v_t,$$

where $v_t = (Gain_t)^\theta - (Loss_t)^\theta$. For the unselected decks, the weights are controlled by the following equations:

$$Explore_{t+1}(d) = Explore_t(d) + \alpha \cdot (\phi - Explore_t(d))$$

and

$$Exploit_{t+1}(d) = \Delta \cdot Exploit_t(d).$$

Appendix B.4. RLWM

The RLWM model is improved upon previously published versions [4, 32] by the inclusion of a choice stickiness parameter. The model has 6 parameters in total: α (learning rate), $bias$ (for negative learning), ϕ (stickiness), ρ (working memory weight), γ (forgetting rate), and K (working memory capacity). The softmax inverse temperature parameter is fixed at $\beta = 20$.

On trial t, the probability of choosing an action a_t in state s_t is given by a weighted combination between a reinforcement learning policy and a working memory one:

$$P(a_t|s_t) = (1 - w) \cdot P_{RL}(a_t|s_t) + w \cdot P_{WM}(a_t|s_t),$$

where $w = \rho \cdot \min(1, \frac{K}{NS})$ and NS is the set size. The action values for both policies are computed as follows:

$$P_{RL}(a_t|s_t) = \frac{\exp\Big(\beta \cdot \big(Q_t(s_t, a_t) + \phi \cdot \mathbb{1}_{a_{t-1}}[a_t]\big)\Big)}{\sum_i \exp\Big(\beta \cdot \big(Q_t(s_t, a_i) + \phi \cdot \mathbb{1}_{a_{t-1}}[a_i]\big)\Big)}$$

and

$$P_{WM}(a_t|s_t) = \frac{\exp\Big(\beta \cdot \big(WM_t(s_t, a_t) + \phi \cdot \mathbb{1}_{a_{t-1}}[a_t]\big)\Big)}{\sum_i \exp\Big(\beta \cdot \big(WM_t(s_t, a_i) + \phi \cdot \mathbb{1}_{a_{t-1}}[a_i]\big)\Big)},$$

where $\mathbb{1}_{a_{t-1}}[a_i]$ is an indicator that takes on the value of 1 if $a_i = a_{t-1}$ and 0 otherwise.

bioRxiv preprint doi: https://doi.org/10.1101/2023.06.19.545524; this version posted January 26, 2024. The copyright holder for this preprint (which was not certified by peer review) is the author/funder, who has granted bioRxiv a license to display the preprint in perpetuity. It is made available under aCC-BY 4.0 International license.

All working memory values are forgotten on each trial:

$$WM_{t+1} = WM_t + \gamma \cdot \Big(\frac{1}{|A|} - WM_t\Big),$$

where $|A|$ is the total number of available actions. The values are then updated according to the following equations:

$$Q_{t+1}(s_t, a_t) = Q_t(s_t, a_t) + \alpha_{RL} \cdot (r_t - Q_t(s_t, a_t))$$

and

$$WM_{t+1}(s_t, a_t) = WM_t(s_t, a_t) + \alpha_{WM} \cdot (r_t - WM_t(s_t, a_t)),$$

where if $r_t = 1$, $\alpha_{RL} = \alpha$ and $\alpha_{WM} = 1$, and if $r_t = 0$, $\alpha_{RL} = bias \cdot \alpha$ and $\alpha_{WM} = bias$.

Appendix B.5. 2-step

The 2-step model [33] contains 6 free parameters: α (learning rate), β_{MB} (softmax inverse temperature for the model-based policy), β_{MF} (softmax inverse temperature for the model-free policy), β (softmax inverse temperature for the second stage), p (stimulus stickiness), and ϕ (response stickiness).

The first-stage decision is made according to action probabilities computed using both the model-based and model-free action values:

$$P(a_t^1) = \frac{\exp\Big(\beta_{MB} \cdot Q_{MB}(a_t^1) + \beta_{MF} \cdot Q_{MF}(a_t^1) + \phi \cdot \mathbb{1}_{a_{t-1}^1}[a_t^1]\Big)}{\sum_i \exp\Big(\beta_{MB} \cdot Q_{MB}(a_i^1) + \beta_{MF} \cdot Q_{MF}(a_i^1) + \phi \cdot \mathbb{1}_{a_{t-1}^1}[a_i^1]\Big)},$$

where $\mathbb{1}_{a_{t-1}^1}[a_i^1]$ is an indicator that takes on the value of 1 if $a_i^1 = a_{t-1}^1$ and 0 otherwise. The second-stage action probabilities are also computed through the softmax function:

$$P(a_t^2|s_t^2) = \frac{\exp\Big(\beta \cdot Q_2(s_t^2, a_t^2)\Big)}{\sum_i \exp\Big(\beta \cdot Q_2(s_t^2, a_i^2)\Big)}.$$

Once the reward r_t has been observed, the action values are updated as

bioRxiv preprint doi: https://doi.org/10.1101/2023.06.19.545524; this version posted January 26, 2024. The copyright holder for this preprint (which was not certified by peer review) is the author/funder, who has granted bioRxiv a license to display the preprint in perpetuity. It is made available under aCC-BY 4.0 International license.

follows:

$$Q_{MF}(a_t^1) \leftarrow Q_{MF}(a_t^1) + \alpha \cdot \Big(Q_2(s_t^2, a_t^2) - Q_{MF}(a_t^1)\Big) + p \cdot \alpha \cdot \Big(r_t - Q_2(s_t^2, a_t^2)\Big)$$

and

$$Q_2(s_t^2, a_t^2) \leftarrow Q_2(s_t^2, a_t^2) + \alpha \cdot \Big(r_t - Q_2(s_t^2, a_t^2)\Big).$$

Note that the model-based action values do not need to be updated and can be computed directly:

$$Q_{MB}(a_t^1) \leftarrow \sum_i \max_j \big(Q_2(s_i^2, a_j^2)\big) \cdot T_{a_t^1}^{s_i^2},$$

where $T_{a_t^1}^{s_i^2}$ is the transition probability from the first-stage choice a_t^1 to the second-stage state s_i^2, which the agent is assumed to know.
